# Supplementary material for: Feasibility trial of a new digital training package to enhance primary care practitioners’ communication of clinical empathy and realistic optimism
Source: PLoS One. 2025 Jul 18;20(7):e0324649. doi: 10.1371/journal.pone.0324649 (PMC12273914; doi:10.1371/journal.pone.0324649)
Supplement: S8. File — (PDF) [file pone.0324649.s008.pdf]

S8 Appendix: Bespoke Osteoarthritis Pain Medication Questionnaire

A bespoke Osteoarthritis Pain Medication Questionnaire was used to assess medication change in the OA group. This instrument, adapted from the validated Medication Change Questionnaire[45] to reduce complexity for patients, asks patients to list all the osteoarthritis pain medications they are using (to include all tablets, medicines, gels, and creams) and to rate any changes in use since starting the study. The main adaptations were: removing the questions "Is there any of this medication that you would like to cut down, to take less of?" and "Is there any of this medication that you would like to take more of?" and the associated details; replacing daily dosage of all medications with a qualitative judgment on any changes in medication use ("For each of these medications, please tell us whether the amount you use has changed since you saw the doctor/nurse/physiotherapist and joined this study approximately two weeks ago. Think about how much you used this before you joined the study. Now think about how much you have use this since you joined the study, about 2 weeks ago. Have you used this medication more, less, or about the same as you did before?" Response options: Much more/A bit more/About the same/A little bit less/Much less).
