# Supplementary material for: Feasibility trial of a new digital training package to enhance primary care practitioners’ communication of clinical empathy and realistic optimism
Source: PLoS One. 2025 Jul 18;20(7):e0324649. doi: 10.1371/journal.pone.0324649 (PMC12273914; doi:10.1371/journal.pone.0324649)
Supplement: S10. Table — (PDF) [file pone.0324649.s010.pdf]

S10. Table.

Baseline scale scores by retained/lost to follow-up

	Lost to follow-up			Retained			Independent t-test		
	N	M	SD	N	M	SD	t	df	p
Age	171	58.52	16.64	206	60.14	15.25	-0.982	375	0.163
Average pain intensity in past week	82	5.99	2.31	107	6.06	2.16	-0.209	187	0.417
Symptom bothersomeness in past week	165	3.83	1.34	207	3.86	1.14	-0.192	370	0.424
Overall Satisfaction (MISS-21)	68	90.12	15.42	96	88.18	14.68	0.817	162	0.208
Enablement (PEI)	108	3.48	1.31	205	3.44	1.20	0.25	311	0.401
Quality of Life - Physical (SF-12)	52	41.92	14.31	136	40.88	12.69	0.487	186	0.313
Quality of Life - Mental (SF-12)	52	44.65	12.57	136	44.77	11.54	-0.064	186	0.474
Wellbeing (Short Warwick Edinburgh Wellbeing Scale)	110	23.57	5.94	203	23.88	5.07	-0.484	311	0.314
Perceived clinician empathy (CARE)	113	37.97	12.43	141	38.43	12.77	-0.288	252	0.387
Perceived clinician optimism	126	5.10	1.24	207	5.13	1.22	-0.162	331	0.436
Treatment Expectancy (CEQ)	58	7.69	2.97	146	7.48	2.87	0.472	202	0.319
Treatment Credibility (CEQ)	60	20.28	6.22	145	19.70	6.30	0.602	203	0.274
Anxiety (HADS-A)	54	9.94	2.80	132	9.26	2.54	1.624	184	0.053
Depression (HADS-D)	54	9.02	1.74	131	9.06	1.84	-0.145	183	0.442
