# Supplementary material for: Feasibility trial of a new digital training package to enhance primary care practitioners’ communication of clinical empathy and realistic optimism
Source: PLoS One. 2025 Jul 18;20(7):e0324649. doi: 10.1371/journal.pone.0324649 (PMC12273914; doi:10.1371/journal.pone.0324649)
Supplement: S11. Table — (PDF) [file pone.0324649.s011.pdf]

S11. Table.

Baseline characteristics by retained/lost to follow-up

Characteristic	Category	Lost to follow-up	Retained
		N(%)	N(%)
Gender [a]	male	39 (51.3%)	37 (48.7%)
	female	125 (42.5%)	169 (57.5%)
Employment [a]	Full time	23 (39.0%)	36 (61.0%)
	Part time	10 (23.8%)	32 (76.2%)
	Retired	42 (29.2%)	102 (70.8%)
	Other	21 (36.2%)	37 (63.8%)
Highest educational qualification [b]	School level or below	47 (40.2%)	70 (59.8%)
	Degree	32 (29.4%)	77 (70.6%)
	Postgraduate degree	13 (23.2%)	43 (76.8%)
Index of Multiple Deprivation [a]	1 (most deprived) – 5	28 (31.1%)	62 (68.9%)
	6 - 10 (least deprived)	55 (31.8%)	118 (68.2%)
First appointment with clinician [a]	yes	61 (44.2%)	77 (55.8%)
	no	107 (45.3%)	129 (54.7%)
Consulted for pain [a]	yes	80 (43.5%)	104 (56.5%)
	no	74 (42.0%)	102 (58.0%)
Consulted for hip or knee OA [a]	yes	14 (34.1%)	27 (65.9%)
	no	146 (44.1%)	185 (55.9%)
Clinician recommended treatment [a]	yes	81 (36.0%)	144 (64.0%)
	no	43 (42.2%)	59 (57.8%)

Note. Z tests compared proportion of participants retained in each category for each characteristic. [a] $p>.05$. [b] $p<.05$
